# Expert consensus on the diagnosis and treatment of solid tumors with BRAF mutations

**DOI:** 10.1016/j.xinn.2024.100661

**Published:** 2024-10-18

**Authors:** Wenxian Wang, Bin Lian, Chunwei Xu, Qian Wang, Ziming Li, Nan Zheng, Aijun Liu, Jinpu Yu, Wenzhao Zhong, Zhijie Wang, Yongchang Zhang, Jingjing Liu, Shirong Zhang, Xiuyu Cai, Anwen Liu, Wen Li, Lili Mao, Ping Zhan, Hongbing Liu, Tangfeng Lv, Liyun Miao, Lingfeng Min, Yu Chen, Jingping Yuan, Feng Wang, Zhansheng Jiang, Gen Lin, Long Huang, Xingxiang Pu, Rongbo Lin, Weifeng Liu, Chuangzhou Rao, Dongqing Lv, Zongyang Yu, Xiaoyan Li, Chuanhao Tang, Chengzhi Zhou, Junping Zhang, Junli Xue, Hui Guo, Qian Chu, Rui Meng, Xuewen Liu, Jingxun Wu, Rui Zhang, Jin Zhou, Zhengfei Zhu, Yongheng Li, Hong Qiu, Fan Xia, Yuanyuan Lu, Xiaofeng Chen, Jian Feng, Rui Ge, Enyong Dai, Yu Han, Weiwei Pan, Fei Pang, Xin Huang, Meizhen Hu, Qing Hao, Kai Wang, Fan Wu, Binbin Song, Bingwei Xu, Liping Wang, Youcai Zhu, Li Lin, Yanru Xie, Xinqing Lin, Jing Cai, Ling Xu, Jisheng Li, Xiaodong Jiao, Kainan Li, Jia Wei, Huijing Feng, Lin Wang, Yingying Du, Wang Yao, Xuefei Shi, Xiaomin Niu, Dongmei Yuan, Yanwen Yao, Jianhui Huang, Yue Feng, Yinbin Zhang, Pingli Sun, Hong Wang, Mingxiang Ye, Dong Wang, Zhaofeng Wang, Yue Hao, Zhen Wang, Bin Wan, Donglai Lv, Shengjie Yang, Jin Kang, Jiatao Zhang, Chao Zhang, Wenfeng Li, Jianfei Fu, Lizhi Wu, Shijie Lan, Juanjuan Ou, Lin Shi, Zhanqiang Zhai, Yina Wang, Bihui Li, Zhang Zhang, Ke Wang, Xuelei Ma, Zhongwu Li, Zhefeng Liu, Nong Yang, Lin Wu, Huijuan Wang, Gu Jin, Guansong Wang, Jiandong Wang, Hubing Shi, Meiyu Fang, Yong Fang, Yuan Li, Xiaojia Wang, Jing Chen, Yiping Zhang, Xixu Zhu, Yi Shen, Shenglin Ma, Biyun Wang, Yong Song, Zhengbo Song, Wenfeng Fang, Yuanzhi Lu, Lu Si

**Affiliations:** 1 Department of Chemotherapy, Chinese Academy of Sciences University Cancer Hospital (Zhejiang Cancer Hospital), Hangzhou, Zhejiang 310022, P.R. China; 2 Key Laboratory of Carcinogenesis and Translational Research (Ministry of Education/Beijing), Department of Melanoma and Sarcoma, Peking University Cancer Hospital & Institute, Beijing 100142, P.R. China; 3 Institute of Cancer and Basic Medicine (ICBM), Chinese Academy of Sciences, Hangzhou, Zhejiang 310022, P.R. China; 4 Department of Respiratory Medicine, Affiliated Jinling Hospital, Medical School of Nanjing University, Nanjing, Jiangsu 210002, P.R. China; 5 Department of Respiratory Medicine, Affiliated Hospital of Nanjing University of Chinese Medicine, Jiangsu Province Hospital of Chinese Medicine, Nanjing, Jiangsu 210029, P.R. China; 6 Department of Shanghai Lung Cancer Center, Shanghai Chest Hospital, Shanghai Jiao Tong University, Shanghai 200030, P.R. China; 7 Beijing Key Laboratory of Mental Disorders, National Clinical Research Center for Mental Disorders & National Center for Mental Disorders, Beijing Anding Hospital, Capital Medical University, Beijing 200030, China; 8 Advanced Innovation Center for Human Brain Protection, Capital Medical University, Beijing 200030, China; 9 Senior Department of Pathology, the 7^th^ Medical Center of PLA General Hospital, Beijing 100700, P.R. China; 10 Department of Cancer Molecular Diagnostics Core, Tianjin Medical University Cancer Institute and Hospital, Tianjin 300060, P.R. China; 11 Guangdong Lung Cancer Institute, Guangdong Provincial Laboratory of Translational Medicine in Lung Cancer, Guangdong Provincial People’s Hospital, Guangdong Academy of Medical Sciences, School of Medicine, Guangzhou, Guangdong 510080, P.R. China; 12 State Key Laboratory of Molecular Oncology, Department of Medical Oncology, National Cancer Center/National Clinical Research Center for Cancer/Cancer Hospital, Chinese Academy of Medical Sciences and Peking Union Medical College, Beijing 100021, P.R. China; 13 Department of Medical Oncology, Lung Cancer and Gastrointestinal Unit, Hunan Cancer Hospital/The Affiliated Cancer Hospital of Xiangya School of Medicine, Central South University, Changsha, Hunan 410013, P.R. China; 14 Department of Thoracic Cancer, Jilin Cancer Hospital, Jilin, Changchun 130012, P.R. China; 15 Translational Medicine Research Center, Key Laboratory of Clinical Cancer Pharmacology and Toxicology Research of Zhejiang Province, Affiliated Hangzhou First People’s Hospital, Cancer Center, West Lake University School of Medicine, Hangzhou, Zhejiang 310006, P.R. China; 16 Department of VIP Inpatient, Sun Yat-Sen University Cancer Center, State Key Laboratory of Oncology in South China, Collaborative Innovation Center for Cancer Medicine, Guangzhou, Guangdong 510060, P.R. ChinaP.R. China; 17 Department of Oncology, Second Affiliated Hospital of Nanchang University, Nanchang, Jiangxi 330006, P.R. China; 18 Key Laboratory of Respiratory Disease of Zhejiang Province, Department of Respiratory and Critical Care Medicine, Second Affiliated Hospital of Zhejiang University School of Medicine, Cancer Center, Zhejiang University, Hangzhou, Zhejiang 310009, P.R. China; 19 Department of Respiratory Medicine, Affiliated Drum Tower Hospital, Medical School of Nanjing University, Nanjing, Jiangsu 210008, P.R. China; 20 Department of Respiratory Medicine, Clinical Medical School of Yangzhou University, Subei People’s Hospital of Jiangsu Province, Yangzhou, Jiangsu 225001, P.R. China; 21 Department of Medical Oncology, Fujian Medical University Cancer Hospital & Fujian Cancer Hospital, Fuzhou, Fujian 350014, P.R. China; 22 Department of Pathology, Renmin Hospital of Wuhan University, Wuhan, Hubei 430060, P.R. China; 23 Department of Internal Medicine, Cancer Center of PLA, Qinhuai Medical Area, Affiliated Jinling Hospital, Medical School of Nanjing University, Nanjing, Jiangsu 210002, P.R. China; 24 Derpartment of Integrative Oncology, Tianjin Medical University Cancer Institute and Hospital, Tianjin 300060, P.R. China; 25 Department of Medical Oncology, Lung Cancer and Hunan Cancer Hospital/The Affiliated Cancer Hospital of Xiangya School of Medicine, Central South University, Changsha, Hunan 410013, P.R. China; 26 Department of Orthopaedic Oncology Surgery, Beijing Ji Shui Tan Hospital, Peking University, Beijing 100035, P.R. China; 27 Department of Radiotherapy and Chemotherapy, Hwamei Hospital, University of Chinese Academy of Sciences, Ningbo, Zhejiang 315010, P.R. China; 28 Department of Pulmonary Medicine, Taizhou Hospital of Wenzhou Medical University, Taizhou, Zhejiang 317000, P.R. China; 29 Department of Respiratory Medicine, the 900^th^ Hospital of the Joint Logistics Team (the Former Fuzhou General Hospital), Fujian Medical University, Fuzhou, Fujian 350025, P.R. China; 30 Department of Oncology, Beijing Tiantan Hospital, Capital Medical University, Beijing 100700, P.R. China; 31 Department of Medical Oncology, Peking University International Hospital, Beijing 102206, P.R. China; 32 State Key Laboratory of Respiratory Disease, National Clinical Research Center for Respiratory Disease, Guangzhou Institute of Respiratory Health, The First Affiliated Hospital of Guangzhou Medical University, Guangzhou, Guangdong 510300, P.R. China; 33 Department of Thoracic Oncology, Shanxi Academy of Medical Sciences, Shanxi Bethune Hospital, Taiyuan, Shanxi 030032, P.R. China; 34 Department of Oncology, Shanghai East Hospital, School of Medicine, Tongji University, Shanghai 200123, P.R. China; 35 Department of Medical Oncology, The First Affiliated Hospital of Xi’an Jiaotong University, Xi’an, Shaanxi 710061, P.R. China; 36 Department of Oncology, Tongji Hospital of Tongji Medical College, Huazhong University of Science and Technology, Wuhan, Hubei 430030, P.R. China; 37 Cancer Center, Union Hospital, Tongji Medical College, Huazhong University of Science and Technology, Wuhan, Hubei 430022, P.R. China; 38 Department of Oncology, the Third Xiangya Hospital, Central South University, Changsha, Hunan 410013, P.R. China; 39 Department of Medical Oncology, the First Affiliated Hospital of Medicine, Xiamen University, Xiamen, Fujian 361003, P.R. China; 40 Department of Medical Oncology, Cancer Hospital of China Medical University, Shenyang, Liaoning 110042, P.R. China; 41 Department of Medical Oncology, Sichuan Cancer Hospital & Institute, Sichuan Cancer Center, School of Medicine, University of Electronic Science and Technology, Chengdu, Sichuan 610041, P.R. China; 42 Department of Radiation Oncology, Fudan University Shanghai Cancer Center, Shanghai 200032, P.R. China; 43 Key Laboratory of Carcinogenesis and Translational Research (Ministry of Education/Beijing), Department of Radiation Oncology, Peking University Cancer Hospital & Institute, Beijing 100142, P.R. China; 44 State Key Laboratory of Cancer Biology, National Clinical Research Center for Digestive Diseases and Xijing Hospital of Digestive Diseases, Fourth Military Medical University, Xi’an, Shaanxi 710032, P.R. China; 45 Department of Oncology, Jiangsu Province Hospital and Nanjing Medical University First Affiliated Hospital, Nanjing, Jiangsu 210029, P.R. China; 46 Department of Respiratory Medicine, Affiliated Hospital of Nantong University, Nantong, Jiangsu 226001, P.R. China; 47 Department of General Surgery, Huadong Hospital Affiliated to Fudan University, Shanghai 200040, P.R. China; 48 Department of Oncology and Hematology, China-Japan Union Hospital of Jilin University, Changchun, Jilin 13003, P.R. China; 49 Department of Gastrointestinal Oncology, Harbin Medical University Cancer Hospital, Harbin, Heilongjiang 1550081, P.R. China; 50 Department of Cell Biology, College of Medicine, Jiaxing University, Jiaxing, Zhejiang 314001, P.R. China; 51 Department of Medical, Shanghai OrigiMed Co., Ltd., Shanghai 201114, P.R. China; 52 Department of Medical, Menarini Silicon Biosystems Spa, Shanghai 400000, P.R. China; 53 Department of Medical Oncology, The Affiliated Hospital of Jiaxing University, Jiaxing, Zhejiang 314000, P.R. China; 54 Department of Biotherapy, Cancer Institute, First Affiliated Hospital of China Medical University, Shenyang 110001, P.R. China; 55 Department of Oncology, Baotou Cancer Hospital, Baotou, Inner Mongolia 014000, P.R. China; 56 Department of Thoracic Disease Diagnosis and Treatment Center, Zhejiang Rongjun Hospital, The Third Affiliated Hospital of Jiaxing University, Jiaxing, Zhejiang 314000, P.R. China; 57 Department of Oncology, Lishui Municipal Central Hospital, Lishui, Zhejiang 323000, P.R. China; 58 Department of Interventional Pulmonary Diseases, Anhui Chest Hospital, Hefei, Anhui 230011, P.R. China; 59 Department of Medical Oncology, Qilu Hospital, Cheeloo College of Medicine, Shandong University, Jinnan, Shangdong 250012, P.R. China; 60 Department of Medical Oncology, Shanghai Changzheng Hospital, Naval Medical University, Shanghai 200070, P.R. China; 61 Department of Oncology, Shandong Provincial Third Hospital, Cheeloo College of Medicine, Shandong University, Jinan, Shandong 250031, P.R. China; 62 Department of the Comprehensive Cancer Center, Affiliated Drum Tower Hospital, Medical School of Nanjing University, Nanjing, Jiangsu 210008, P.R. China; 63 Department of Pathology, Shanxi Academy of Medical Sciences, Shanxi Bethune Hospital, Taiyuan, Shanxi 030032, P.R. China; 64 Department of Oncology, The First Affiliated Hospital of Anhui Medical University, Hefei, Anhui 230022, P.R. China; 65 Department of Interventional Oncology, The First Affiliated Hospital, Sun Yat-sen University, Guangzhou, Guangdong 510060, P.R. China; 66 Department of Respiratory Medicine, Huzhou Hospital, Zhejiang University School of Medicine, Huzhou, Zhejiang 313000, P.R. China; 67 Department of Gynecologic Radiation Oncology, Chinese Academy of Sciences University Cancer Hospital (Zhejiang Cancer Hospital), Hangzhou, Zhejiang 310022, P.R. China; 68 Department of Oncology, the Second Affiliated Hospital of Medical College, Xi’an Jiaotong University, Xi’an, Shaanxi 710004, P.R. China; 69 Department of Pathology, The Second Hospital of Jilin University, Changchun, Jilin 130041, P.R. China; 70 Senior Department of Oncology, The 5^th^ Medical Center of PLA General Hospital, Beijing 100071, P.R. China; 71 Department of Radiation Oncology, Affiliated Jinling Hospital, Medical School of Nanjing University, Nanjing, Jiangsu 210002, P.R. China; 72 Department of Respiratory Medicine, The Affiliated Jiangning Hospital of Nanjing Medical University, Nanjing, Jiangsu 210002, P.R. China; 73 Department of Clinical Oncology, The 901 Hospital of Joint Logistics Support Force of People Liberation Army, Hefei, Anhui 230031, P.R. China; 74 Department of Thoracic Surgery, Chuxiong Yi Autonomous Prefecture People’s Hospital, Chuxiong, Yunnan 675000, P.R. China; 75 Department of Radiation Oncology, First Affiliated Hospital of Wenzhou Medical College, Wenzhou, Zhejiang 325000, China; 76 Department of Medical Oncology, Affiliated Jinhua Hospital, Zhejiang University School of Medicine, Jinhua, Zhejiang 321000, P.R. China; 77 Department of Microsurgery, Taizhou Hospital Affiliated to Wenzhou Medical University, Taizhou, Zhejiang 317000, China; 78 Department of Cancer Center, The First Hospital of Jilin University, Changchun, Jilin 130021, P.R. China; 79 Department of Oncology and Southwest Cancer Center, Southwest Hospital, Third Military Medical University (Army Medical University), Chongqing 400038, P.R. China; 80 Department of Respiratory Medicine, Zhongshan Hospital, Fudan University, Shanghai 200032, P.R. China; 81 Department of Oncology, The First Affiliated Hospital, College of Medicine, Zhejiang University, Hangzhou, Zhejiang 310000, P.R. China; 82 Department of Oncology, The Second Affiliated Hospital of Guilin Medical University, Guilin, Guangxi 541199, P.R. China; 83 International Cooperative Laboratory of Traditional Chinese Medicine Modernization and Innovative Drug Discovery of Chinese Ministry of Education (MOE), Guangzhou City Key Laboratory of Precision Chemical Drug Development, School of Pharmacy, Jinan University, Guangzhou, Guangdong 510632, P.R. China; 84 National Health Commission (NHC) Key Laboratory of Nuclear Medicine, Jiangsu Key Laboratory of Molecular Nuclear Medicine, Jiangsu Institute of Nuclear Medicine, Wuxi 214063, China; 85 Department of Radiopharmaceuticals, School of Pharmacy, Nanjing Medical University, Nanjing, Jiangsu 210000, People's Republic of China; 86 Department of Biotherapy, State Key Laboratory of Biotherapy, Cancer Center, West China Hospital, Sichuan University, Chengdu, Sichuan 610041, P.R. China; 87 Key Laboratory of Carcinogenesis and Translational Research (Ministry of Education/Beijing), Department of Pathology, Peking University Cancer Hospital & Institute, Beijing 100142, P.R. China; 88 Department of Internal Medicine, The Affiliated Cancer Hospital of Zhengzhou University, Henan Cancer Hospital, Zhengzhou, Henan 450000, P.R. China; 89 Department of Bone and Soft-tissue Surgery, Chinese Academy of Sciences University Cancer Hospital (Zhejiang Cancer Hospital), Hangzhou, Zhejiang 310022, P.R. China; 90 Institute of Respiratory Diseases, Xinqiao Hospital, Third Military Medical University, Chongqing 400037, P.R. China; 91 Department of Pathology, Affiliated Jinling Hospital, Medical School of Nanjing University, Nanjing, Jiangsu 210002, P.R. China; 92 Frontier Science Center for Disease Molecular Network, West China Hospital, Sichuan University, Chengdu, Sichuan 610041, P.R. China; 93 Department of Medical Oncology, Sir Run Run Shaw Hospital, Zhejiang University, Hangzhou, Zhejiang 310016, P.R. China; 94 Department of Pathology, Fudan University Shanghai Cancer Center, Shanghai 200032, P.R. China; 95 Department of Thoracic Surgery, Affiliated Jinling Hospital, Medical School of Nanjing University, Nanjing, Jiangsu 210002, P.R. China; 96 Department of Oncology, Key Laboratory of Clinical Cancer Pharmacology and Toxicology Research of Zhejiang Province, Affiliated Hangzhou Cancer Hospital, Cancer Center, Zhejiang University School of Medicine, Hangzhou, Zhejiang 310006, P.R. China; 97 Department of Breast Cancer and Urological Medical Oncology, Fudan University Shanghai Cancer Center, Department of Oncology, Shanghai Medical College, Fudan University, Shanghai 200032, P.R. China; 98 Department of Medical Oncology, Sun Yat-sen University Cancer Center, State Key Laboratory of Oncology in South China, Collaborative Innovation Center for Cancer Medicine, Guangzhou, Guangdong 510060, P.R. China; 99 Department of Clinical Pathology, The First Affiliated Hospital of Jinan University, Guangzhou, Guangdong 510630, P.R. China

**Keywords:** solid tumors, tyrosine receptor kinase, precision medicine, targeted therapy, BRAF mutation

## Abstract

The BRAF gene is an important signaling molecule in human cells that is involved in the regulation of cell growth, differentiation, and survival. When the BRAF gene mutates, it can lead to abnormal activation of the signaling pathway, which promotes cell proliferation, inhibits cell apoptosis, and ultimately contributes to the occurrence and development of cancer. BRAF mutations are widely present in various cancers, including malignant melanoma, thyroid cancer, colorectal cancer, non-small cell lung cancer, and hairy cell leukemia, among others. BRAF is an important target for the treatment of various solid tumors, and targeted combination therapies, represented by BRAF inhibitors, have become one of the main treatment modalities for a variety of BRAF-mutation-positive solid tumors. Dabrafenib plus trametinib, as the first tumor-agnostic therapy, has been approved by the US Food and Drug Administration for the treatment of adult and pediatric patients aged 6 years and older harboring a BRAF V600E mutation with unresectable or metastatic solid tumors that have progressed following prior treatment and who have no satisfactory alternative treatment options. This is also the first time a BRAF/MEK inhibitor combination has been approved for use in pediatric patients. As research into the diagnosis and treatment of BRAF mutations advances, standardizing the detection of BRAF mutations and the clinical application of BRAF inhibitors becomes increasingly important. Therefore, we have established a universal and systematic strategy for diagnosing and treating solid tumors with BRAF mutations. In this expert consensus, we (1) summarize the epidemiology and clinical characteristics of BRAF mutations in different solid tumors, (2) provide recommendations for the selection of genetic testing methods and platforms, and (3) establish a universal strategy for the diagnosis and treatment of patients with solid tumors harboring BRAF mutations.

## Public summary

• BRAF is an important signaling molecule in human cells.
• Mutations in *BRAF* facilitate the occurrence and development of cancer.
• *BRAF* mutations common in various cancers, representing a significant therapeutic target for multiple solid tumors.
• *BRAF* mutation detection and BRAF inhibitors’ clinical application have become increasingly significant.

## Graphical abstract

**Figure:**
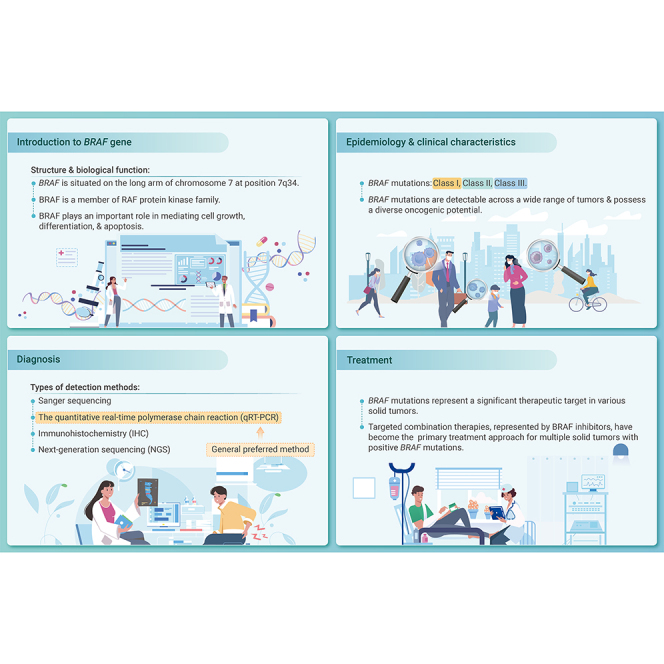

## Introduction

The BRAF gene is a key component of the mitogen-activated protein kinase (MAPK) signaling pathway, regulating cell growth, differentiation, and survival. Mutations in the BRAF gene can lead to persistent activation of the pathway, resulting in uncontrolled cell proliferation and tumor development.^1^^,^^2^ Mutations in the BRAF gene can be detected in a variety of tumors and have broad-spectrum carcinogenicity. They are commonly found in solid tumors such as malignant melanoma, thyroid cancer, colorectal cancer, and non-small cell lung cancer (NSCLC) and are occasionally seen in gliomas, cholangiocarcinomas, gastrointestinal stromal tumors, and others.^3^^,^^4^^,^^5^

The most common mutation in the BRAF gene is the V600E mutation, which accounts for the majority of BRAF mutations across various tumor types. In addition, other less common mutations have been identified, such as V600K, V600R, and fusion mutations.^6^^,^^7^^,^^8^^,^^9^ By detecting BRAF gene mutations, more precise treatment options can be provided for patients. For instance, melanoma patients with the BRAF V600E mutation may benefit from targeted therapy drugs aimed at the BRAF protein, such as vemurafenib (brand name: Zelboraf) and dabrafenib (brand name: Tafinlar)^10^; in colorectal cancer patients, the regimen of encorafenib (brand name: Braftovi) combined with cetuximab (brand name: Erbitux) has been approved in the United States for use in patients with metastatic colorectal cancer (mCRC) with the BRAF V600E mutation who have previously undergone treatment and whose disease has progressed.^11^ Therefore, accurate detection of BRAF mutations is essential for selecting appropriate targeted therapies, and various methods can be used, including PCR,^12^^,^^13^^,^^14^^,^^15^ immunohistochemistry (IHC),^16^ next-generation sequencing (NGS),^12^^,^^17^ and RNA-based NGS to detect BRAF mutations.^18^^,^^19^^,^^20^^,^^21^ Different detection methods have their advantages and limitations.

The clinical application of BRAF-targeted therapy has completely transformed the treatment of solid tumors with BRAF mutations. Currently, several anti-tumor drugs targeting BRAF mutations have been approved for the market. For example, dabrafenib in combination with trametinib is used for the treatment of patients with BRAF-mutation-positive metastatic NSCLC^22^; dabrafenib plus trametinib is used for adjuvant treatment after surgery in patients with BRAF-V600-mutation-positive stage III melanoma^23^; vemurafenib is used for the treatment of patients with BRAF-V600-mutation-positive advanced melanoma,^24^ and so forth. However, the same drug may exhibit differences across various tumor types. Furthermore, resistance to the medication may develop over time after administration.^25^^,^^26^

International experts have reached a consensus on the diagnosis and treatment of solid tumors with BRAF mutations, providing valuable insights into the biology, classification, clinical characteristics, and detection methods of BRAF mutations. This consensus also emphasizes the clinical application of BRAF-targeted therapy across different types of tumors. It also provides a comprehensive guide for clinicians in the diagnosis and treatment of solid tumors with BRAF mutations.

## The biological basis of the BRAF gene

### The gene structures and biological functions of the BRAF gene

BRAF, also known as v-Raf murine sarcoma viral oncogene homolog B1, is located on the long arm of chromosome 7 (7q34) and is one of the members of the family that encodes RAF protein kinases. Other members of this family include ARAF and CRAF (RAF1).^27^ RAF protein kinases are part of the RAS-RAF-MEK-ERK signaling pathway (also known as the MAPK/ERK pathway).

The upstream regulation of BRAF primarily involves the KRAS and EGFR pathways. Regarding the EGFR pathway, EGFR (epidermal growth factor receptor) is a receptor tyrosine kinase located on the cell surface, which can be activated by various ligands, such as the epidermal growth factor (EGF). Upon ligand binding, EGFR undergoes dimerization, leading to the activation of its internal tyrosine kinase domain, followed by autophosphorylation. The activation of EGFR triggers multiple intracellular signaling pathways, including the PI3K-Akt pathway and the RAS-RAF-MEK-ERK pathway. Regarding the KRAS pathway, KRAS is a small guanosine triphosphatase (GTPase), which is activated when bound to GTP and inactivated when bound to guanosine diphosphate. The activation of EGFR can promote the GTP binding of KRAS through a series of adaptor proteins, thereby activating KRAS. The activated KRAS is able to directly interact with downstream RAF kinases (including ARAF, BRAF, and CRAF), promoting their activation.^28^^,^^29^

Downstream, the regulation mediated by BRAF primarily functions through MEK and ERK to link cell-surface receptors with transcription factors in the cell nucleus, thereby activating various factors involved in regulating numerous biological events within the cell, such as cell growth, differentiation, and apoptosis.^1^ Under normal circumstances, RAF proteins control the activity of downstream signaling pathways and cell growth according to the signals they receive.^30^ However, when BRAF undergoes pathogenic mutations, it can lead to the RAF proteins being in a state of constant activation, continuously transmitting signals to downstream pathways even without receiving signals, thereby causing cells to grow and proliferate uncontrollably.^2^^,^^31^

### The classes of BRAF mutations

**Figure 1:**
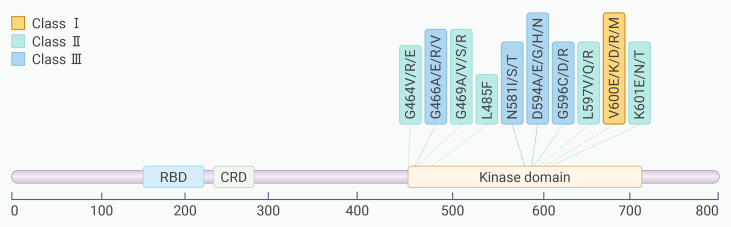
Frequency and location of BRAF hotspots in solid tumor

Based on whether the mutation has kinase activity and whether the kinase activity is dependent on RAS signaling and RAF dimerization, BRAF mutations can be classified into class I, class II, and class III mutations (Table 1 and Figure 1).^31^

**Table 1:** Details of class I, II, and III BRAF mutations

BRAF mutation	Mutation details
Class I BRAF mutation	BRAFV600E, BRAFV600K, BRAFV600R, BRAFV600D
Class II BRAF mutation	the active segment of the kinase domain (K601 E/N/T, L597V/Q/R), P-ring (G464 V/E, G469 A/V/R)
Class III BRAF mutation	P-ring (G466 A/E/V), catalytic ring (N581I/S/T), DFG motif (D594 A/G/H/N, D596D/R) of kinase domain
Deletion	kinase domain α-C-spiral short segment missing whole frame
Fusion	N-end loss

Class I BRAF mutations, namely BRAF V600 mutations, account for over 90% of BRAF variations in cancer. BRAF V600 mutations can be observed in approximately 50% of melanoma patients, 40% of papillary thyroid carcinoma patients, and 10% of colorectal cancer patients.^32^ Among these, BRAF V600E is the most common amino acid substitution, accounting for over 90% of BRAF V600 mutations. In other instances, the valine (V) at codon 600 in exon 15 may also be substituted by other amino acids, such as lysine, arginine, or aspartic acid.^32^ In lung and colorectal cancer patients who have acquired resistance to EGFR inhibitors, class I BRAF mutations are often detected.^33^^,^^34^ Class I BRAF mutations can lead to aberrant activation of BRAF kinase and spontaneous, sustained activation of the MAPK signaling pathway. Class I mutations activate downstream signaling pathways as monomers and do not depend on RAS activity, showing sensitivity to BRAF inhibitors as well as downstream MEK inhibitors or a combination of both.^35^

Non-V600 BRAF mutations can be divided into two types based on their signaling characteristics and dependence on RAS activation: class II BRAF mutations and class III BRAF mutations. Class II BRAF mutations are often located in the active segment of the kinase domain (such as K601 E/N/T, L597V/Q/R) or the P loop (such as G464 V/E and G469 A/V/R), exhibit moderate or high kinase activity, activate downstream signaling pathways as dimers, and do not depend on RAS activity. In class II BRAF mutants, high ERK activation drives a feedback mechanism that activates RAS,^36^ and class II BRAF mutations typically do not co-occur with other alterations in the MAPK pathway. Class II BRAF mutations are sensitive to BRAF dimer inhibitors, pan-RAF inhibitors, MEK inhibitors, or a combination of these agents. In patients with NSCLC carrying EGFR mutations, class II BRAF mutations are often considered an important mechanism for the acquired resistance to EGFR inhibitors.^37^

Class III BRAF mutations are typically located in the kinase domain’s P loop (such as G466 A/E/V), the catalytic loop (such as N581I/S/T), and the DFG motif (such as D594 A/G/H/N and D596D/R). They exhibit lower kinase activity than wild-type BRAF or are kinase impaired and amplify upstream activation signals by forming heterodimers with CRAF or wild-type BRAF, requiring dependence on RAS activity.^6^^,^^7^^,^^8^^,^^9^ Class III BRAF mutations often occur in tumors with high receptor tyrosine kinase (RTK) activity and are frequently accompanied by RAS activation or loss of NF1 function.^38^ The frequency of RAS and NF1 mutations varies depending on the tumor tissue of origin: in melanoma, due to the lower endogenous baseline RAS activity, class III BRAF mutations almost always coexist with mutated RAS or NF1. In colorectal and lung cancers, due to their higher baseline RTK activity, the resulting RAS activity is sufficient to support the activation of class III BRAF mutants; therefore, only a minority of class III BRAF-mutation cases coexist with RAS/NF1 mutations.^38^^,^^39^ In the absence of concurrent RAS or NF1 mutations, the growth of tumors with class III BRAF mutations is sensitive to the inhibition of RTKs (which can drive RAS activity).^38^ Therefore, class III BRAF mutations are associated with increased sensitivity to EGFR inhibitors and prolonged survival after treatment in patients with colorectal cancer,^38^^,^^40^^,^^41^ a phenomenon that is in stark contrast to class I BRAF mutations, as colorectal cancer patients with class I mutations respond poorly to EGFR inhibitors and have less favorable survival outcomes after treatment.

In addition to point mutations, BRAF also harbors rare variant types such as deletions and fusions. Loss of BRAF activation typically involves in-frame deletions of short segments in the kinase domain’s α-C helix, leading to enhanced kinase activity. Such variants do not depend on RAS activity and are mechanistically similar to class II mutations.^35^^,^^42^^,^^43^ BRAF deletions are present in approximately 0.5%–1% of patients with pancreatic and thyroid cancers^43^^,^^44^ and account for about 5% in patients with KRAS wild-type pancreatic cancer.^43^

Activating BRAF fusions typically includes the complete kinase domain, and the loss of the N terminus results in the loss of the autoinhibitory mechanism, leading to the formation of active BRAF dimers that activate downstream signaling pathways. Mechanistically, BRAF fusions are similar to class II mutations.^6^ BRAF fusions account for approximately 0.3% of all cancers, about 3%–4% in melanomas, around 0.3% in pancreatic cancers, and approximately 20% in KRAS wild-type pancreatic cancers and pancreatic acinar cell carcinomas, while in pilocytic astrocytomas they represent over 50%.^45^^,^^46^^,^^47^^,^^48^

### Clinical characteristics and prevalence of BRAF mutations in different cancer types

BRAF gene mutations can be detected in a variety of tumors and have a broad-spectrum oncogenic potential. They are commonly found in solid tumors such as malignant melanoma, thyroid cancer, colorectal cancer, and NSCLC, and are occasionally observed in gliomas, cholangiocarcinomas, and gastrointestinal stromal tumors, among others,^3^^,^^4^ as detailed in Table 2.

**Table 2:** Summary of BRAF-mutation rates in different tumor types

Tumor type	Cancer type	BRAF-mutation rate	Explanation of the BRAF V600 mutation site
Tumors of the central neural system	glioblastoma multiforme	1.70%	BRAF V600E
	ganglioglioma	43%	BRAF V600E
	pleomorphic xanthoastrocytoma	66%	BRAF V600E
	pilocytic astrocytoma	70%–80%	BRAF V600E (10%)，mostly BRAF-KIAA1549 fusion
	pediatric low-grade astrocytoma	30%	BRAF V600E
	pediatric high-grade astrocytoma	15%	BRAF V600E
	adult low-grade astrocytoma	10%	BRAF V600E
	adult high-grade astrocytoma	3%	BRAF V600E
	craniopharyngioma	81%	BRAF V600E
	Langerhans cell histiocytosis	25%–38%	BRAF V600E
	Erdheim-Chester disease	54%	BRAF V600E
Melanoma	melanoma	∼60%	BRAF V600E (80%); BRAF V600K (8%); BRAF V600R (1%); other codons (10%)
Head and neck/oral and maxillofacial tumors	anaplastic thyroid carcinoma	30%–80%	BRAF V600E
	papillary thyroid carcinoma	44%	BRAF V600E
	ameloblastoma	70.00%	BRAF V600E
	salivary gland tumor	3%–13%	–
Respiratory system tumors	non-small cell lung cancer (NSCLC)	1%–5%	BRAF V600E (50%)
Digestive system tumors	gastrointestinal stromal tumor	2%–13%	BRAF V600E
	cholangiocarcinoma	3%–22%	BRAF V600E (60%); BRAF V600D (13%); other codons (27%)
	pancreatic cancer	1%–16%	BRAF V600E
	colorectal cancer	5%–15%	BRAF V600E
	pyloric gland adenoma	12%	–
Reproductive system tumors	ovarian cancer	35%–60%	BRAF V600E
Urinary system tumors	renal cancer	3%	BRAF V600E (85%); other codons (5%)
	prostate cancer	1.60%	BRAF V600E (<1%); BRAF V600X (84%)

#### Melanoma

In Western populations, patients with melanoma are predominantly affected by the cutaneous type, with BRAF gene mutations accounting for 50%–60%, of which over 90% are class I V600 mutations. More than 90% of these are BRAF V600E (encoding GTG>GAG), followed by BRAF V600K, which represents 5%–6%; the remainder include BRAF V600R (GTG>AGG), BRAF V600 “E2” (GTG>GAA), and BRAF V600D (GTG>GAT).^49^ In East Asian populations, patients with melanoma are primarily affected by acral and mucosal types, with a BRAF-mutation frequency of around 15%–25%, of which V600E accounts for over 80%.^50^^,^^51^ With increasing age, the incidence of BRAF mutations decreases; tumors in patients over the age of 70 have mutations in 25% of cases, while tumors in patients under the age of 30 almost universally exhibit BRAF mutations. Additionally, V600E mutations are more commonly found in skin intermittently exposed to sunlight, particularly in superficial spreading melanomas, whereas non-V600E mutations are more common in areas of chronic sun exposure, such as the head and neck.^52^^,^^53^

#### Thyroid cancer

Kimura et al.^54^ first reported the presence of BRAF mutations in 35.8% (28/78) of differentiated thyroid cancers in 2003. Nikiforova et al.^55^ were the first to report that BRAF mutations were confined to patients with papillary thyroid carcinoma (PTC) or to those with poorly differentiated or anaplastic thyroid cancers arising from previous PTC. Furthermore, a pooled analysis of data from 29 studies showed that BRAF V600E mutations occurred in 44% of patients with PTC and 24% of patients with anaplastic thyroid carcinoma (ATC).^56^ Although rare non-V600 mutations have been reported, the majority of mutations in PTC or ATC are BRAF V600 mutations.^57^

#### Non-small cell lung cancer

BRAF mutations occur in approximately 1%–5% of NSCLC cases, are associated with adenocarcinoma (89.3% vs. 70.6%, *p* = 0.048), and are more prevalent in never-smokers (78.6% vs. 56.7%, *p* = 0.019).^58^ Among these, class I BRAF V600 mutations are the predominant type, accounting for approximately 30%–50%, with the V600E mutation being the most common (about 90%) and frequently occurring in micropapillary adenocarcinoma.^59^^,^^60^^,^^61^ The study by Awad and colleagues^7^ included 236 patients with NSCLC carrying BRAF mutations, of which class I mutations accounted for approximately 45% (107 cases), class II mutations for 32% (75 cases), and class III mutations for 23% (54 cases). BRAF V600 mutations may be more common in patients with a light or no smoking history, while non-V600 BRAF mutations are often found in heavy smokers.^62^

#### Colorectal cancer

In colorectal cancer patients, BRAF mutations account for approximately 4%–12% of all colorectal cancer cases,^63^ and about 90% of these mutations are V600E mutations.^64^ BRAF-mutant colorectal cancer is more common in female patients aged ≥70 years or in those with a smoking history, and it is associated with a high rate of peritoneal and distant lymph node metastases and a low rate of lung metastases.^65^^,^^66^^,^^67^^,^^68^ BRAF gene mutations are significantly more prevalent in colon cancer than in rectal cancer, are associated with a lower level of differentiation, tend to be mucinous, and often accompany MSI-H (microsatellite instability—high) molecular alterations.^69^^,^^70^^,^^71^^,^^72^

## Types of detection methods and their limitations

### Sanger sequencing

Sanger sequencing is a classic direct sequencing technique that determines DNA sequences in FFPE (formalin-fixed, paraffin-embedded) and cytological specimens to identify both known and unknown mutations in the BRAF gene. Sanger sequencing allows for the identification of all BRAF mutations. It should be noted that although Sanger sequencing is a reliable method, it has a lower sensitivity and can only detect variants with a mutation rate higher than 15%. Moreover, because it can only sequence one DNA fragment at a time, it is a time-consuming method.^12^^,^^13^^,^^17^^,^^73^ For the detection of complex gene mutations or low-frequency mutations, other more sensitive genetic testing techniques are required (Table 3).^12^^,^^13^

**Table 3:** Comparison of different detection methods

Detection method	Advantages	Shortcomings
Sanger sequencing	allows recognition of all BRAF mutations	low sensitivity and time consuming
qPCR	high sensitivity, short reporting cycle, relatively low cost, strong convenience, and easy implementation in hospitals. Recommended as the preferred conventional method for detecting BRAF mutations	only applicable for detecting known mutations
IHC	suitable for small samples, easy to explain, low cost, high efficiency, and can reveal the existence and distribution characteristics of heterogeneous tumor cells. It has gradually become a commonly used method for detecting BRAF gene mutations	it can specifically detect BRAF V600E mutant proteins but cannot simultaneously detect other types of mutant proteins, such as V600K
NGS	higher sensitivity (approximately 0.001%–5% of mutation allele frequencies relative to wild-type allele backgrounds) and the ability to detect a wide range of BRAF mutations	conventional diagnosis faces many challenges, such as high costs and turnaround time, as well as the need for specialized knowledge in bioinformatics

### Quantitative real-time PCR

Real-time fluorescence quantitative PCR (qPCR) can qualitatively and quantitatively measure BRAF mutations in tumor tissues, cytological samples, or liquid biopsy specimens such as blood, pleural effusion, and bronchoalveolar lavage fluid. This method has the advantages of high sensitivity, short reporting cycle, relatively low cost, convenience, and ease of implementation within hospitals. For qPCR, upstream and downstream primers need to be designed based on known mutation sites; therefore, qPCR is only suitable for detecting known mutations. Additionally, compared to Sanger sequencing, qPCR is more sensitive in detecting the BRAF V600E mutation, with sensitivity sufficient to detect mutations in mixtures containing as little as 0.001% mutant DNA, whereas the detection limit for Sanger sequencing is 15%.^14^^,^^15^ Given its advantages, qPCR can be recommended as the preferred routine method for the detection of BRAF mutations (Table 3).

### Immunohistochemistry

IHC is a method that uses specific antibodies to identify the BRAF V600E mutant protein for the detection of BRAF mutations. It has high concordance with Sanger sequencing and PCR methods, can be used even with small tumor samples, is easy to interpret, and offers the advantages of being both inexpensive and efficient. It has gradually become a commonly used method for detecting BRAF gene mutations.^16^ Comparative analysis of tissue samples from patients with BRAF mutations across multiple cancer types using Sanger sequencing, qPCR, and IHC has confirmed that IHC (using the VE1 monoclonal antibody) has sensitivity and specificity of 100% and 99%, respectively, for the BRAF V600E mutation.^74^ Based on the high sensitivity and specificity of the VE1 antibody, the National Comprehensive Cancer Network guidelines recommend the following for BRAF V600E IHC testing: VE1 can be used for large-scale preliminary screening to determine the BRAF V600E mutation status in melanoma; in colorectal cancer, IHC testing for BRAF V600E can be used for predicting treatment efficacy and prognostic assessment in patients with BRAF mutations, guiding medication, and aiding in the diagnosis of Lynch syndrome; in thyroid cancer, IHC testing can be used for differential diagnosis and guiding medication; in lung cancer, IHC can detect BRAF V600E, but widespread implementation requires laboratory validation. Furthermore, the international expert consensus on the diagnosis and treatment of BRAF mutations in NSCLC explicitly recommends IHC as a supplementary screening method. Current studies in China comparing the concordance between IHC and molecular testing for BRAF mutations in NSCLC show that, compared to molecular testing, IHC (using the VE1 antibody) has sensitivity of 97.2%, specificity of 100.0%, and concordance rate of 99.2%.^75^ Therefore, the BRAF V600E mutation can be detected in Chinese NSCLC patients using the IHC method (VE1 antibody).

Additionally, the positive intensity varies among patients, with most laboratories quantifying it as 0 (negative), 1+ (weakly positive), 2+ (moderately positive), and 3+ (strongly positive).^16^^,^^76^^,^^77^ Furthermore, a weak VE1-positive signal does not have a significant correlation with the presence of a BRAF mutation, as IHC samples showing positive intensities ranging from 1+ to 3+ can also be detected with mutations in Sanger sequencing.^76^^,^^78^ Moreover, tumor cells with BRAF mutations exhibit heterogeneous staining in IHC.^79^^,^^80^ Given that sequencing technologies require a relatively high degree of homogeneity and content of mutant DNA, IHC can reveal the presence and distribution characteristics of heterogeneous tumor cells, which helps in better selection of sequencing areas and increases its sensitivity.

Additionally, VE1, as a monoclonal antibody for detecting the V600E mutation, has high specificity and does not cross-react with non-V600E sites of BRAF.^81^^,^^82^ Therefore, IHC can specifically detect the BRAF V600E mutant protein, but it cannot detect other types of mutant proteins such as V600K simultaneously. The targeted drugs dabrafenib in combination with trametinib have therapeutic effects on BRAF V600 mutations (BRAF V600 E/K/D/R/M).^83^ If patients are only tested for the V600E mutation using IHC, there is a possibility that other types of mutations may be missed. Therefore, it cannot completely replace sequencing but can serve as a complement to other sequencing methods (Table 3).

### Next-generation sequencing

#### DNA-based and RNA-based NGS

NGS is a high-throughput DNA-sequencing technology that can sequence DNA or RNA rapidly and accurately. Compared to first-generation sequencing technologies, NGS has significantly increased sequencing speed and throughput while reducing costs. NGS technology allows whole-genome sequencing, whole-exome sequencing, and targeted sequencing of thousands of genes in a single run for tumor tissues, cytological samples, or liquid biopsy samples such as blood, pleural effusions, and bronchoalveolar lavage fluids. Compared to other methods, it has higher sensitivity (about 0.001%–5% mutant allele frequency relative to the wild-type allele background) and can detect a wide range of BRAF mutations. However, implementing NGS in routine diagnostics faces several challenges, such as high costs and turnaround time, as well as the need for expertise in bioinformatics (Table 3).

(1) DNA-based NGS detection technology. NGS testing targets DNA or RNA. DNA-based NGS directly sequences DNA to obtain genetic information. At the DNA level, fusion breakpoints typically occur within longer intronic regions and exhibit diversity across different patients. Using NGS-based methods to design probes for capturing the breakpoints is a feasible detection approach. However, detecting fusion genes at the DNA level inevitably involves the following limitations and challenges: (1) to accurately locate the fusion breakpoints, comprehensive coverage of the exceedingly lengthy and repetitive-sequence-rich intronic regions is required for fusion gene detection; (2) variations in GC content within intronic regions are not conducive to the uniform and effective capture of target region fragments by probes; (3) introns of different genes contain very similar repetitive sequences, a characteristic that is not conducive to accurate sequence alignment, affecting the precision of detection; and (4) complex transcriptional or post-transcriptional splicing processes may affect the detection of fusion genes.^84^
(2) RNA-based NGS detection technology. According to the central dogma, mature RNA is produced through transcription and splicing processing using DNA as a template.^18^ RNA-based NGS first transcribes RNA into cDNA, and the sequencing data obtained reflects the gene-expression levels at a specific point in time. RNA-based NGS has the potential to overcome the shortcomings of DNA-NGS. Probes used for RNA-based NGS only cover exons, making probe design less challenging than for DNA, and can detect gene-fusion variants formed at the transcriptional level through splicing. In cases where DNA-based NGS does not detect fusion genes, RNA-based NGS can increase the detection rate of fusion genes.^85^^,^^86^ RNA-based NGS testing often utilizes FFPE tissue samples, and RNA degradation resulting from long-term storage of these samples has become one of the concerns in the clinical setting.^87^ The NGS library construction process includes methods such as amplicon-based and hybrid-capture-based approaches. Compared to hybrid-capture-based methods, amplicon-based methods require a lower input of nucleic acid templates and have a shorter library construction time, which can to some extent reduce the impact of RNA degradation on the detection results.^19^^,^^20^^,^^21^ In patients with driver gene negativity based on DNA-based NGS testing, RNA-based NGS can detect an additional 10%–14.2% of actionable fusion variants.^88^^,^^89^

#### Others

(1) Tissue NGS test. Tissue NGS test typically refers to the high-throughput sequencing technology applied to DNA or RNA from tissue samples. This method enables researchers to rapidly and accurately analyze the genetic information within tissue samples, including gene-expression patterns, genetic variations, mutations, and more. Currently, tissue NGS testing remains the gold standard for determining BRAF mutations in tumors. However, the accuracy of tissue testing is not 100%, as tumors exhibit heterogeneity; mutations carried by tumor tissues can vary at different stages and from different locations within the tumor. Additionally, for some patients whose tumors are too small for biopsy, liquid biopsy can serve as an alternative method when tissue samples cannot be obtained.^90^^,^^91^^,^^92^^,^^93^
(2) Circulating tumor DNA (ctDNA). ctDNA testing is a liquid biopsy technology designed to detect tumor-derived biomarkers by analyzing free DNA in body fluids such as blood. It is rapid and minimally invasive and can reflect the spatial and temporal heterogeneity of the tumor.^94^^,^^95^ As found in the study by Thierry et al.,^96^ ctDNA BRAF V600E mutation analysis in colorectal cancer can achieve 100% concordance with tissue analysis. However, results obtained from ctDNA must be interpreted with caution. The main limitations include: (1) limited diagnostic sensitivity in patients with low tumor burden; (2) differentiation between free non-tumor cell DNA and tumor cell DNA; (3) lack of information on histological types; and (4) inability to validate by IHC.^97^ Therefore, ctDNA-NGS can currently serve as a complement to tissue NGS testing, providing additional genetic information.
(3) CTC-NGS. NGS testing of circulating tumor cells (CTCs) is also a non-invasive method used to detect the presence and characteristics of circulating tumor cells in a patient’s blood.^98^ NGS testing of CTCs involves capturing and isolating CTCs, followed by molecular biological analysis of these cells to determine the presence of mutations in the BRAF gene. This approach allows for the acquisition of information on tumor mutations through blood samples without the need for tissue biopsy or surgical excision.^99^ The advantage of CTC-NGS lies primarily in its non-invasive nature, which can avoid the risks and discomfort associated with traditional tissue biopsies. Second, CTC-NGS can be collected and analyzed at different time points, providing real-time monitoring of the dynamic changes in BRAF mutations in the tumor. Lastly, NGS testing of CTCs can address the issues of insufficient sensitivity and susceptibility to interference from white blood cell background mutations found in ctDNA testing, serving as a powerful complementary diagnostic tool to ctDNA testing.^100^^,^^101^^,^^102^

It should be noted that, when considering NGS as a routine diagnostic selection method, factors such as tissue availability, DNA quality, tumor cell percentage, sensitivity and specificity of various tests, workload, hands-on time, and turnaround time, as well as the cost of testing and equipment and the professional interpretation of test results, need to be taken into account.^93^^,^^97^

## The clinical application of BRAF mutations

BRAF mutations are one of the important therapeutic targets for various solid tumors, and targeted combination therapies, represented by BRAF inhibitors, have become a major treatment modality for a variety of BRAF-mutation-positive solid tumors. Currently, approved BRAF inhibitors are primarily categorized into specific BRAF inhibitors and non-specific BRAF inhibitors based on their targets of action. Specific BRAF inhibitors include dabrafenib, vemurafenib, and encorafenib. Non-specific BRAF inhibitors include sorafenib, regorafenib, pazopanib, and donafenib. The current list of first-line drug regimens approved by the US Food and Drug Administration (FDA) for various cancer types can be found in Table 4. Some mechanisms of resistance to BRAF-targeted therapy can be found in Table 5.^103^^,^^104^^,^^105^^,^^106^

**Table 4:** List of FDA-approved recommended drugs for various BRAF-mutation cancers

Cancer types	Treatment stage	Recommended drugs
Unresectable/metastatic melanoma	first-line treatment	dabrafenib + trametinib
		vemurafenib + cobimetinib
		vemurafenib + cobimetinib + atezolizumab
		encorafenib + binimetinib
Stage III melanoma	adjuvant therapy	dabrafenib + trametinib
Unresectable/metastatic lung cancer	first-line treatment	dabrafenib + trametinib
Unresectable/metastatic colorectal cancer	following treatment with one or two forms of non-EGFR antibody treatments	encorafenib + cetuximab
Unresectable/metastatic anaplastic thyroid cancer	first-line treatment	dabrafenib + trametinib
Unresectable/metastatic pan-cancer types (age 6 and older, such as glioma, cholangiocarcinoma, gastrointestinal stromal tumor, cancer of unknown primary, non-colorectal cancer)	second-line or above treatment	dabrafenib + trametinib

**Table 5:** Summary of drug-resistance mechanisms in partial BRAF-targeted therapy

Cancer species	Medicine	Drug-resistance mechanism
NSCLC	combination therapy of dabrafenib and trametinib	acquired mutations in NRAS p.Q61R and amplification of NTRK
NSCLC	dabrafenib, vemurafenib	PDGFRβ overexpression
NSCLC (adenocarcinoma subtype)	combination therapy of dabrafenib and trametinib	KRAS G12V mutation
Diffuse intrinsic pontine glioma (DIPG)	trametinib	MEK1 or MEK2 mutations
Melanoma	dabrafenib, vemurafenib	PI3K/AKT pathway activation
Colorectal cancer	cetuximab	upregulation of PD-L1 expression activates the PI3K/AKT signaling pathway
Melanoma	vemurafenib, dabrafenib	MITF amplification
NSCLC	combination therapy of dabrafenib and trametinib	increased expression of PDGFRβ and IGFR1

### Treatments for melanoma patients with BRAF mutations

The primary treatment for resectable melanoma is surgical excision.^107^ To improve overall survival rates, adjuvant therapies for postoperative melanoma and systemic treatment for advanced melanoma, such as immunotherapy and targeted therapy, are often employed.^108^^,^^109^

#### Dabrafenib monotherapy and dabrafenib in combination with trametinib therapy

In 2013, the FDA approved dabrafenib as a single-agent treatment for adult patients with unresectable or metastatic melanoma carrying the BRAF V600E mutation.^110^ In 2015, the European Union approved the combination of dabrafenib and the MEK inhibitor trametinib ("D + T" regimen) for the treatment of adult patients with unresectable or metastatic melanoma positive for the BRAF V600 E/K mutation.^83^ In 2018, the FDA approved the combination of dabrafenib and trametinib for adjuvant treatment in patients with melanoma harboring BRAF V600 E/K mutations.^111^ In 2022, the FDA granted accelerated approval for the combination of dabrafenib and trametinib for the treatment of adult and pediatric patients aged 6 years and older with unresectable or metastatic solid tumors harboring the BRAF V600E mutation who have progressed following prior treatment and who have no satisfactory alternative treatment options. Currently, the approved indications for the combination of dabrafenib and trametinib in China are: (1) for patients with unresectable or metastatic melanoma positive for the BRAF V600 mutation; (2) as adjuvant therapy following complete resection in patients with stage III melanoma positive for the BRAF V600 mutation; and (3) for patients with metastatic NSCLC positive for the BRAF V600 mutation. All three approved indications have been included in the national medical insurance drug list.

#### Vemurafenib monotherapy and vemurafenib in combination with cobimetinib therapy

In 2011, the FDA approved vemurafenib for the treatment of adult patients with BRAF V600E mutation in unresectable or metastatic melanoma.^112^ In 2015, the FDA approved the combination of vemurafenib and the MEK inhibitor cobimetinib for the treatment of unresectable or metastatic melanoma with BRAF V600E or V600K mutations.^113^ In 2020, the FDA approved the first immune-combined dual-targeted triple-drug regimen, vemurafenib in combination with cobimetinib plus the programmed death ligand 1 (PD-L1) inhibitor atezolizumab, for first-line treatment of patients with BRAF-V600-mutation-positive, unresectable, or metastatic melanoma.^114^ Currently, the approved indication for vemurafenib monotherapy in China is for unresectable or metastatic melanoma positive for the BRAF V600 mutation as determined by a China FDA-approved testing method.

#### Encorafenib in combination with binimetinib therapy

In 2018, the FDA approved the combination of encorafenib and binimetinib for first-line treatment of patients with unresectable or metastatic melanoma with BRAF V600 E/K mutations.^115^

### Treatments for colorectal cancer patients with BRAF mutations

Treatment options for colorectal cancer include the eradication of potential infections, surgery, cryosurgery, chemotherapy, radiotherapy, and targeted therapy,^116^ with surgery being the primary form of treatment, often supplemented by chemotherapy.

The primary treatment for patients with RAS wild type and BRAF wild type is anti-EGFR monoclonal antibodies combined with chemotherapy; for patients with RAS mutations and BRAF wild type, the main treatment is chemotherapy (based on fluoropyrimidine doublet or triplet chemotherapy) combined with the anti-VEGF drug bevacizumab.^117^

Currently, for first-line treatment of advanced colorectal cancer with BRAF V600E mutation, both domestic and international guidelines recommend chemotherapy in combination with bevacizumab as the standard treatment. In a phase 2 randomized study, FOLFOXIRI combined with bevacizumab or cetuximab was utilized as a first-line treatment regimen for BRAF V600E mutant mCRC. The results showed that the response rates for the cetuximab group versus the bevacizumab group were 50.8% and 66.7%, respectively. This outcome indicates that in the first-line treatment of RAS wild-type/BRAF V600E mutant mCRC, FOLFOXIRI + bevacizumab demonstrates better efficacy compared to FOLFOXIRI + cetuximab.^118^

If first-line treatment fails, the overall treatment strategy for second-line therapy in colorectal cancer patients with BRAF V600E mutation is primarily based on targeted drugs. Research has shown that the combination of dabrafenib and trametinib demonstrates meaningful efficacy in various BRAF-positive tumor types, such as NSCLC, melanoma, and ATC, and among patients with some rare cancers that lack other treatment options. However, this therapy is not suitable for BRAF-mutant colorectal cancer.^119^ This is due to the reactivation of the MAPK pathway mediated by the EGFR, leading to treatment resistance.^120^ An open-label, randomized, phase 2 study aimed to evaluate the efficacy of cetuximab + irinotecan ± vemurafenib in treating BRAF-V600E-mutated colorectal cancer. The results showed that cetuximab + irinotecan + vemurafenib compared to cetuximab + irinotecan had a progression-free survival (PFS) of 4.4 months vs. 2.0 months, an objective response rate (ORR) of 17% vs. 4%, and an overall survival (OS) of 9.6 months vs. 5.9 months.^121^ Based on the study results, the Chinese Society of Clinical Oncology (CSCO) guidelines recommend cetuximab + irinotecan + vemurafenib for second-line and beyond treatment in patients with RAS wild-type/BRAF V600E mutation. In 2023, the CSCO recommended the use of BRAF inhibitors + cetuximab ± MEK inhibitors for second-line and beyond treatment in patients with RAS wild-type/BRAF V600E mutations.

In the BEACON study, 665 patients with BRAF-V600E-mutant mCRC who had progressed after one or two prior treatments were enrolled. Patients were randomized in a 1:1:1 ratio to receive doublet therapy (encorafenib combined with the EGFR monoclonal antibody cetuximab), triplet combination targeted therapy (encorafenib + binimetinib + cetuximab), or one of the two control therapies (cetuximab combined with irinotecan or the cetuximab + FOLFIRI regimen). Results showed that the doublet therapy with encorafenib and cetuximab had a longer PFS (4.2 vs. 4.3 vs. 1.5 months) and higher ORR (20% vs. 26% vs. 2%) as well as the lowest rate of adverse events (50% vs. 58% vs. 61%).^122^ In 2020, the FDA approved the combination therapy of encorafenib and cetuximab for the treatment of patients with mCRC harboring BRAF V600E mutations who have progressed after one or two prior treatments.^122^

### Treatments for lung cancer patients with BRAF mutations

#### Treatment of patients with BRAF V600E mutation

BRAF is an oncogenic driver that can activate the RAS-RAF-MEK-ERK pathway and induce cell growth and excessive proliferation.^123^ Between 2% and 4% of NSCLC patients have BRAF gene mutations, of which approximately 50% are detected as V600E.^124^^,^^125^ A variety of targeted therapies have achieved good efficacy in lung cancers with BRAF mutations.

Dabrafenib, an oral small-molecule inhibitor of BRAF mutations, reduces the phosphorylation levels of MEK and ERK by inhibiting BRAF kinase activity, thereby inhibiting cell proliferation and promoting cell-cycle arrest and cell death.^126^ An open-label, single-arm, multi-center clinical study enrolled 84 patients with stage IV metastatic NSCLC harboring the BRAF V600E mutation. The results demonstrated that after treatment with oral dabrafenib, among the 78 treated patients, the ORR was 33% (95% confidence interval [CI]: 3.4–7.3 months), and the median duration of response was 12.7 months (95% CI: 7.3–16.9 months). In the cohort of six naive patients, four showed an objective response. Out of the 84 patients, 35 (42%) experienced grade 3–4 adverse events, including cutaneous squamous cell carcinoma (12%), fatigue (5%), and basal cell carcinoma (5%).^127^

Trametinib is a MEK1/2 inhibitor that blocks the kinase activity of MEK1/2 and prevents RAF-dependent MEK phosphorylation mediated by BRAF kinase.^128^ Multiple clinical trials have demonstrated the superior therapeutic efficacy of the combination treatment with dabrafenib and trametinib. A Chinese registration clinical study on the combination of dabrafenib and trametinib included 20 patients with BRAF-mutated NSCLC. After treatment with the combination of dabrafenib and trametinib, the ORR was as high as 75% (95% CI: 50.9%–91.3%), with one patient (5%) achieving complete pathological remission, and the disease control rate reached 95% (95% CI: 75.1%–99.9%).^129^ The results of another real-world study showed that among 40 patients with advanced NSCLC harboring the BRAF V600E mutation, the median PFS and median OS for the treatment with dabrafenib combined with trametinib were 17.5 months (95% CI: 7.1–23 months) and 25.5 months (95% CI: 16.6 months—not reached), respectively. This indicates that in the real-world setting, the efficacy of dabrafenib combined with trametinib in patients with advanced NSCLC and BRAF V600 mutation is similar to that observed in previous clinical trials.^22^ Researches above indicate that the dual-targeted therapy regimen of dabrafenib combined with trametinib exhibits good anti-tumor effects in patients with BRAF-V600-mutant NSCLC, whether as a first line or subsequent line of treatment. Currently, the combination of dabrafenib and trametinib has been approved in China for three indications: the treatment of BRAF-V600-mutation-positive metastatic NSCLC, adjuvant treatment after surgery for BRAF-V600-mutation-positive melanoma, and the treatment of unresectable or metastatic melanoma. Numerous authoritative guidelines both domestically and internationally recommend the combination of dabrafenib and trametinib as the preferred treatment for patients with BRAF-V600-mutation-positive NSCLC.

#### Treatment of patients with non-V600 BRAF mutations

In patients with NSCLC, over 50% of BRAF mutations are non-V600 mutations, and real-world studies have shown that some patients with non-V600 BRAF mutations can also benefit from targeted therapy.^130^^,^^131^ For instance, a retrospective multi-center cohort study analyzed advanced lung cancer patients with BRAF mutations who received targeted therapy (vemurafenib, dabrafenib, and sorafenib), and the results showed that among six patients with non-V600E BRAF mutations, one patient (17%) with the BRAF G596V mutation experienced a partial response after treatment with vemurafenib.^132^ Currently there is no standard targeted therapy regimen for non-V600E BRAF mutations, and patients with such genetic mutations respond differently to various BRAF inhibitors. Further large-scale, high-quality research is needed to provide references for clinical treatment plans for these patients.

#### Treatment of EGFR-TKI-resistant patients with BRAF V600 mutation

BRAF mutation is one of the mechanisms of acquired resistance to EGFR-TKI. In 1,637 patients with EGFR mutations who developed resistance after EGFR-TKI treatment, 71 (4.3%, 71/1637) had BRAF mutations.^133^ Current case reports suggest that patients with advanced NSCLC who develop resistance to EGFR-TKI therapy with a concurrent BRAF V600 mutation may benefit from a dual-targeted treatment combined with EGFR-TKI. For example, a 50-year-old man diagnosed with lung adenocarcinoma with EGFR T790M and EGFR E746_A750del mutations experienced disease progression after treatment with osimertinib. A re-biopsy revealed the tumor had BRAF V600 and EGFR E746_A750del mutations. After switching to a combination therapy of dabrafenib, trametinib, and osimertinib for 2 months, a CT scan showed complete remission in the lymph nodes and significant remission in the lungs and bones. Adverse events during treatment were primarily fatigue, dysgeusia, fever, and nausea, with the fever resolving on its own within 2 weeks.^134^

### Other clinical treatment information related to BRAF mutations

In 2018, the FDA approved dabrafenib/trametinib for BRAF-V600E-mutation ATC. In 2022, the FDA granted accelerated approval for dabrafenib/trametinib for the treatment of adult and pediatric patients aged 6 years and older with unresectable or metastatic solid tumors harboring a BRAF V600E mutation who have progressed following prior treatment and who have no satisfactory alternative treatment options. This is the first and only BRAF/MEK inhibitor approved for use in tumors of unknown primary origin carrying the BRAF V600E mutation, and it is also the only BRAF/MEK inhibitor therapy approved for use in pediatrics.

Additionally, for patients with advanced solid tumors harboring a confirmed BRAF V600 mutation, treatment recommendations should be refined based on the specific tumor type and molecular pathological characteristics, such as the presence of MSI-H or high PD-L1 expression.^50^^,^^135^^,^^136^^,^^137^^,^^138^^,^^139^ Apart from melanoma, immunotherapy should be cautiously prioritized.^50^^,^^140^^,^^141^

### Drug-resistance mechanism

In most cases, resistance mechanisms to BRAF inhibitors include reactivation of the MAPK pathway (such as overexpression of NRAS) or activation of alternative RTK-mediated survival pathways (such as overexpression of PDGFRβ).^25^ A 2015 study analyzed 132 samples obtained from tissues of melanoma patients with disease progression following treatment with BRAF inhibitors, identifying potential mechanisms of resistance in 58% of the cases. Among these cases, 20% had NRAS/KRAS mutations, 30% had BRAF mutations or BRAF amplifications, and 7% had MEK1/2 mutations. Additionally, 11% of the cases reported alterations in non-MAPK pathways (PI3K/AKT, MITF amplification, PDGFR/IGF1R upregulation).^26^ MATCH-R (NCT 02517892) is a prospective study on resistance mechanisms to immunotherapy and targeted therapy conducted at the Gustave Roussy Cancer Center in France involving tumor patients who have experienced no progression for more than 6 months following immunotherapy and targeted therapy. From December 2014 to July 2019, 11 patients with BRAF-V600E-mutation NSCLC were included in the MATCH-R study, all diagnosed with adenocarcinoma and negative for EGFR, ALK, ROS1, KRAS, and HER2. Tissue samples from four patients revealed definitive resistance mutations to BRAF/MEK inhibitors, including MEK1 K57N, PTEN N329fs, NRAS Q61R, and KRAS Q61R mutations. Additionally, a KRAS G12V mutation was detected in the ctDNA from the peripheral blood of one patient.^142^ Analysis of ctDNA from blood samples of colorectal cancer patients in the BEACON trial showed that the majority of resistance mechanisms were due to reactivation of the MAPK pathway.^138^

## BRAF mutation and immune microenvironment

In terms of immune regulation, the mechanisms by which BRAF mutations participate in immune modulation are complex and multi-faceted.

### Tumor microenvironment alteration

BRAF mutations can lead to the secretion of various cytokines and chemokines (such as CCL2, CCL5, and CXCL8) by tumor cells, altering the surrounding tumor microenvironment. This attracts immunosuppressive cells (such as regulatory T cells and tumor-associated macrophages) while repelling or inhibiting the infiltration of effector immune cells (such as CD8^+^ T cells).

### Tumor-antigen presentation

BRAF mutations may affect the antigen-processing and -presentation mechanisms of tumor cells, reducing the expression of major histocompatibility complex molecules and weakening the ability of tumor cells to be recognized by the immune system.

### Immune escape

BRAF-mutated tumor cells may inhibit T cell activation and function by expressing immune checkpoint molecules (such as PD-L1), achieving escape from the immune system.

### Cell-signaling pathways

BRAF mutations activate the MAPK/ERK signaling pathway. The activation of the MAPK/ERK pathway is often associated with cell proliferation, cell differentiation, and the production of cytokines. Therefore, BRAF mutations may lead to excessive proliferation of tumor cells and participate in the regulation affecting epithelial-mesenchymal transition, which in turn influences the behavior of immune cells.^143^^,^^144^^,^^145^^,^^146^

### Treatment response

The use of BRAF inhibitors may affect the interaction between tumor cells and the immune system. Recent studies have shown that, in addition to directly targeting tumor cells, BRAF inhibitors may also enhance anti-tumor effects by modulating the immune response within the tumor microenvironment. Specifically, BRAF inhibitors can promote the infiltration of immune cells within the tumor, such as CD8^+^ T cells and natural killer cells, which are crucial for recognizing and killing tumor cells.^147^ Additionally, BRAF inhibitors may downregulate the expression of immune checkpoint molecules on the surface of tumor cells, such as PD-L1, which helps to relieve the immunosuppression imposed by the tumor and enhances the anti-tumor activity of T cells.^148^

Therefore, the combination of BRAF inhibitors with immune checkpoint inhibitors (such as PD-1/PD-L1 antibodies) has emerged as a novel therapeutic strategy. This approach aims to enhance treatment efficacy through a dual mechanism, directly inhibiting tumor growth while also strengthening the anti-tumor response of the immune system. Clinical trials have shown that this combination therapy has good efficacy in certain types of cancer, particularly achieving significant success in the treatment of melanoma. BRAF inhibitors, such as vemurafenib, have been proven as effective for melanomas with BRAF V600 mutations but often come with a shorter duration of response and the potential for developing resistance. On the other hand, immune checkpoint inhibitors such as the PD-L1 antibody atezolizumab can activate the patient’s own immune system to fight cancer cells and, while they may have a relatively lower rate of effectiveness, the duration of response can be longer once they take effect. One clinical trial has demonstrated that atezolizumab combined with vemurafenib produced a high ORR of 75% in patients with untreated BRAF-V600-mutant melanoma, including complete remission in three patients and partial remission in ten patients. This combination therapy performed well in terms of safety, with no dose-limiting toxicities or treatment discontinuations related to atezolizumab.

Furthermore, for BRAF-V600-mutant melanoma patients, the combination therapy of PD-1 and CTLA-4 inhibitors has been approved as a first-line treatment by the FDA and European Medicines Agency.^149^^,^^150^ Recent studies have confirmed the clinical efficacy of combination treatments with BRAF inhibitors and dual immunotherapies. The SECOMBIT trial, a phase 2, randomized, three-arm, non-comparative, open-label study, enrolled patients with unresectable stage III or IV melanoma. The participants were randomized in a 1:1:1 ratio into three groups. Group A (targeted-immune) patients received encorafenib and binimetinib until disease progression (PD), after which they received ipilimumab and nivolumab until secondary PD. Group B (immune-targeted) patients were treated with ipilimumab and nivolumab until PD, following which they received encorafenib and binimetinib until secondary PD. Group C (targeted-immune-targeted) patients received encorafenib and binimetinib for 8 weeks, followed by ipilimumab and nivolumab until PD, then encorafenib and binimetinib until secondary PD. Long-term follow-up results from the SECOMBIT trial showed that the 4-year OS rates were 59% for group B and 63% for group C, while group A had a rate of 46%. This study further solidifies the position of immunotherapy as the preferred first-line treatment for BRAF-V600-mutant metastatic melanoma patients. Additionally, it indicates that patients with markedly impaired basal immunity at baseline may require a temporary application of BRAF/MEK inhibitors to halt rapid disease progression.^151^

### Autophagy and apoptosis

BRAF mutations may also impact the processes of autophagy and apoptosis in tumor cells, which are important for the activation of immune cells and the release of tumor antigens. When a BRAF gene mutation occurs, such as the BRAF V600E mutation, it can lead to sustained aberrant activation of the MAPK signaling pathway, promoting tumor cell proliferation and survival while also affecting cellular autophagy and apoptosis. Autophagy is a self-digestive process that helps cells eliminate damaged components and proteins, maintaining cellular homeostasis. In certain circumstances, autophagy can also promote the occurrence and development of tumors, especially in melanoma with the BRAF V600E mutation, where increased levels of autophagy are associated with the tumor’s resistance to chemotherapy. Moreover, BRAF mutations may lead to tumor cell resistance to chemotherapeutic drugs, as autophagy can protect cancer cells from the cytotoxic effects of chemotherapy.^143^^,^^144^^,^^145^^,^^146^

## Summary and prospects

**Figure 2:**
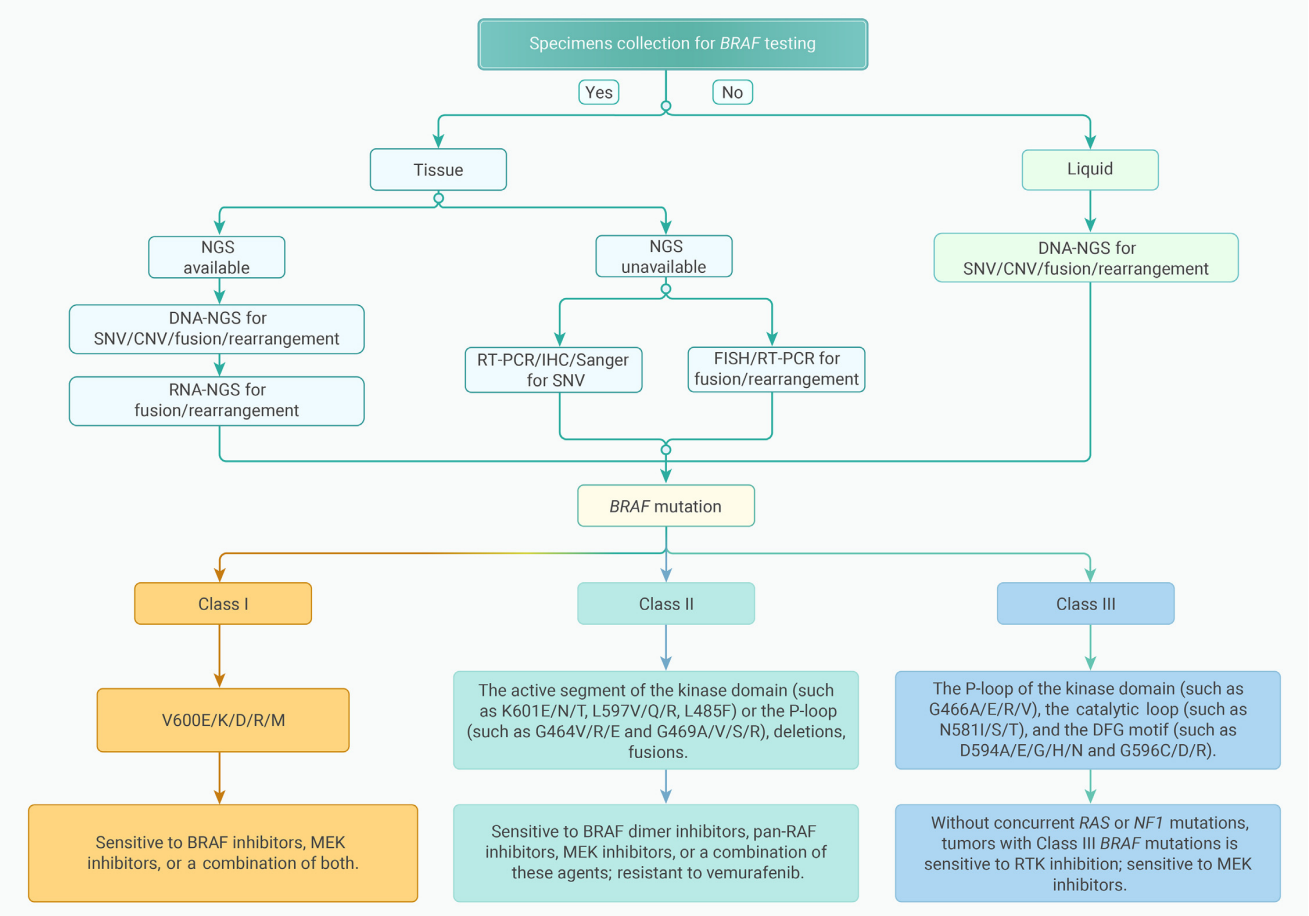
The recommended procedure for the diagnosis and treatment of BRAF gene mutation solid tumors

BRAF mutations are key oncogenic drivers in various solid tumors, particularly in malignant melanoma, thyroid cancer, colorectal cancer, and NSCLC. Under normal circumstances, RAF proteins control the activity of downstream signaling pathways and cell growth according to the signals they receive.^30^ However, when BRAF undergoes pathogenic mutations it can lead to the RAF protein being in a state of persistent activation, continuously transmitting signals to its downstream pathways even without receiving signals, resulting in uncontrolled cell growth and proliferation.^2^^,^^31^ The consensus highlights the importance of BRAF gene mutations in solid tumors and provides detailed information on mutation frequency, mutation types, detection methods, and drug-development progress across various tumor types. It is noteworthy that the emergence of BRAF inhibitors has changed the treatment landscape for these tumors, but there are differences in efficacy and resistance among different types of tumors, which may be due to the varying biological characteristics of the tumors. Therefore, accurate detection of BRAF mutations is crucial for guiding treatment choices. The consensus recommends various detection methods, including PCR, IHC, and NGS, to ensure the precise diagnosis of BRAF mutations. Through the consensus, clinicians can better understand the role of BRAF mutations in tumor development, select the most appropriate detection methods, and provide personalized treatment plans for patients (Table 6 and Figure 2).

**Table 6:** Agreement on the identification and management of solid malignancies with BRAF gene alterations

	Consensus number	Key points	Recommendation level
Detection time point	consensus 1	it is advised to perform BRAF-mutation testing for tumor categories that are sanctioned for therapy with BRAF inhibitors, primarily encompassing melanoma, NSCLC, colorectal carcinoma, and thyroid carcinoma	strongly recommended
	consensus 2	BRAF-mutation testing should be conducted across various cancer types. For patients with point mutations, fusions or rearrangements, it is recommended that they participate in relevant clinical trials	strongly recommended
Detection method	consensus 3	guidance suggests prioritizing BRAF-mutation analysis through NGS, with the scope of mutations examined encompassing single-nucleotide variants, fusions, or rearrangements	strongly recommended
	consensus 4	DNA-based NGS panels and fluorescence *in situ* hybridization (FISH) stand as the principal techniques to identify BRAF rearrangements; meanwhile, RNA-based NGS panels or whole-transcriptome sequencing serve as valuable additional approaches to uncover BRAF rearrangements. Where feasible, the recommendation is to isolate nucleic acids, both deoxyribonucleic acid and ribonucleic acid, from fresh specimens or FFPE (tissues preserved with formalin and encased in paraffin) to enable simultaneous testing using DNA- and RNA-focused NGS techniques	recommended
	consensus 5	NGS-based testing kits ought to explicitly indicate the regions spanned by the probes (encompassing intronic regions), and for the prevention of false-negative results, choosing products with comprehensive probe coverage is advised	recommended
	consensus 6	When NGS is not accessible, it is advised to conduct BRAF-mutation analysis through other techniques (point mutations are identifiable by reverse transcription RT-PCR, IHC, or Sanger method; fusions or structural variations are identifiable by FISH or RT-PCR)	strongly recommended
	consensus 7	RT-PCR can be used as detection methods for known BRAF gene-fusion variants, but for patients with unknown fusions or negative test results, it is recommended to use NGS or FISH as a method for reconfirmation	recommended
Detection strategy	consensus 8	BRAF testing ought to be given precedence within neoplastic tissue specimens (from surgical resections, biopsies, and the like). For patients from whom tumor tissue is not obtainable, liquid biopsy testing (e.g., blood, urine, pleural or peritoneal effusion, bronchoalveolar lavage fluid, cerebrospinal fluid) can be performed, taking into consideration the specific tumor characteristics. Nonetheless, the constraints associated with analyzing these samples ought to be explicitly outlined within the diagnostic report	strongly recommended
	consensus 9	circulating tumor DNA (ctDNA) testing technology is well established and has high sensitivity. It is recommended for patients whose tissue samples are difficult to obtain to undergo ctDNA testing to acquire genomic data of the patient, which can provide reference information for targeted therapy and prognosis	strongly recommended
	consensus 10	it is advisable to conduct genetic testing on individuals previously administered BRAF inhibitors to expand their therapeutic possibilities	strongly recommended
Detecting quality control	consensus 11	for tumor tissues and cytological samples, an experienced pathologist should evaluate the tumor cell content. When carrying out IHC or FISH, a minimum of 50 neoplastic cells is necessary; During the execution of RT-PCR, a minimal neoplastic cell content of 5% is essential; During the application of NGS, it is necessary for the specimen to contain a minimum neoplastic cell proportion of 20%. If the neoplastic cell concentration at the interface is inadequate, microdissection may be contemplated to augment the neoplastic tissue	strongly recommended
	consensus 12	laboratories ought to engage in yearly proficiency testing schemes such as Pathology Quality Control Center, College of American Pathologists, Clinical Laboratory Improvement Amendments, or alternative cross-laboratory quality evaluations; The dependability of testing outcomes may also be verified by juxtaposing them with outcomes from accredited labs employing identical analytical techniques; When results are not in agreement, it is essential to have access to different techniques for confirmation and examination	strongly recommended
	consensus 13	beyond fundamental details and quality assurance metrics, the assay documentation ought to encompass the neoplastic cell percentage, histological condition at the microscopic level, and the extracted DNA’s yield and integrity. For reports from NGS assays, it is essential to detail the precise junction points on the chromosomal regions, whether the tyrosine kinase region is affected, and whether the gene rearrangements maintain the proper reading frame. If a gene rearrangement of BRAF includes the kinase region and preserves the reading frame, it ought to be documented as a gene-fusion event; in other cases, it should be classified and recorded as a genomic alteration	strongly recommended
	consensus 14	in instances where the clinician encounters uncertainties (such as discrepancies across various assays, novel fusion partners or configurations, intricate gene-fusion scenarios, challenges in verifying the continuity of the reading frame or the entirety of the kinase region, or co-occurrence of several oncogenic drivers), consultation with the Molecular Tumor Board is advised to validate subsequent therapeutic strategies	recommended
Treatment strategy	consensus 15	in cases of individuals presenting with late-stage solid malignancies harboring a verified mutation in BRAF V600 and also exhibiting molecular pathological characteristics such as MSI-H or high PD-L1 expression, treatment recommendations should be refined based on the specific type of tumor. Apart from cutaneous melanoma, immunotherapy should be carefully considered as a priority recommendation	strongly recommended
	consensus 16	in individuals diagnosed with progressive NSCLC possessing a validated BRAF V600 alteration, the preferred therapeutic approach involves a dual regimen of BRAF and MEK inhibition (for instance, utilizing dabrafenib alongside trametinib)	strongly recommended
	consensus 17	individuals with advanced or metastatic melanoma and a verified mutation in BRAF V600 should consider therapeutic options that include a combination of BRAF and MEK inhibitors (such as the pairing of dabrafenib with trametinib) or alternative medicinal interventions	strongly recommended
	consensus 18	for progressive colorectal cancer harboring a BRAF V600 alteration, the suggested therapeutic approach is to administer chemotherapeutic agents alongside bevacizumab	strongly recommended
	consensus 19	for patients with BRAF mutations in tumor types or mutation variants that have not been approved, it is recommended that they participate in relevant clinical trials	recommended

Although we currently have a deeper understanding of the role of BRAF mutations in solid tumors and their impact on treatment, the field still faces a series of complex challenges and is fostering new opportunities. At present, the key challenge we face is that the heterogeneity and complexity of BRAF-mutant tumors continue to pose difficulties for the development of individualized treatment strategies. Even tumors in different patients with the same BRAF mutation may have vastly different treatment responses and efficacies. In response to this key challenge, our insight is that future research should focus on more detailed molecular subtype classification and the synergistic effects with mutations in other pathways, thereby developing more precise treatment methods. In summary, this consensus provides comprehensive guidance for the diagnosis and treatment of BRAF mutations in solid tumors, helping to improve treatment outcomes and the quality of life for patients.

## Acknowledgments

This work was supported by the 10.13039/501100001809Natural Science Foundation of China (grant number 82002456), China Postdoctoral Science Foundation (grant number 2022M723207), the 10.13039/501100014759Medical Scientific Research Foundation of Zhejiang Province, China (grant number 2023KY666), Zhejiang Traditional Chinese Medicine Science Fund Project (grant number 2024ZL372), Qiantang Cross Fund Project (grant number 2023-16), National Natural Science Foundation of China of Zhejiang Cancer Hospital Cultivation Project (grant number PY2023006), the 10.13039/501100014759Medical Scientific Research Foundation of Zhejiang Province, China (grant number 2024KY812), and the 10.13039/501100004731Natural Science Foundation of Zhejiang Province (grant number LQ24H160036), Beijing Health Technologies Promotion Program [grant number BHTPP2022041], Peking University Clinical Scientist Training Program and the Fundamental Research Funds for the Central Universities [grant number BMU2024PYJH010], Science Foundation of Peking University Cancer Hospital [grant number PY202333], the Beijing Natural Science Foundation [grant number 7232248] and Beijing Hospitals Authority Youth Programme [grant number QML20231902].

## Author contributions

Z.S., W.F., Yuanzhi Lu, and L. Si participated in the design of the expert consensus. W.W., B. Lian, C.X., and Q.W. conceived the expert consensus, participated in its design and coordination with other authors, and helped to draft the expert consensus. All authors read and approved the final manuscript.

## Declaration of interests

The authors declare no competing interests.

## Lead contact website

The lead contact website is at https://www.researchgate.net/profile/Chunwei-Xu.
